# Drug Intensification in Future Postoperative Radiotherapy Practice in Biochemically-Relapsing Prostate Cancer Patients

**DOI:** 10.3389/fonc.2021.780507

**Published:** 2021-12-24

**Authors:** Axel Cailleteau, Paul Sargos, Fred Saad, Igor Latorzeff, Stéphane Supiot

**Affiliations:** ^1^ Department of Radiation Oncology, Institut de Cancérologie de l’Ouest, Nantes Saint-Herblain, France; ^2^ Department of Radiation Oncology, Institut Bergonié, Bordeaux, France; ^3^ Department of Urology, Université de Montréal, Montreal, QC, Canada; ^4^ Department of Radiation Oncology, Oncorad Clinique Pasteur, Toulouse, France; ^5^ University of Nantes, CRCINA (CNRS, Inserm), Nantes, France

**Keywords:** radiosensitizing agents, PARP inhibitors, androgen receptor (AR) antagonist, combined treatment, salvage prostate bed radiotherapy

## Abstract

Although salvage prostate bed radiotherapy is highly effective in biochemically-relapsing prostate cancer patients following prostatectomy, relapses remain frequent and improvements are needed. Randomized phase 3 trials have shown the benefit of adding androgen-depriving therapy to irradiation, but not all patients benefit from this combination. Preclinical studies have shown that novel agents targeting the androgen receptor, DNA repair, PI3K/AKT/mTOR pathways, or the hypoxic microenvironment may help increase the response to prostate bed irradiation while minimizing potential side effects. This perspective review focuses on the most relevant molecules that may have an impact when combined with salvage radiotherapy, and underlines the strategies that need to be developed to increase the efficacy of salvage post-prostatectomy radiotherapy in prostate cancer patients.

## Introduction

Despite adequate surgery, biochemical relapse following prostatectomy for locally advanced prostate cancer occurs in up to 50% of cases (1). One interesting result of adjuvant radiotherapy trials is that treatment failure is mainly a result of lack of local control (2, 3). This corroborates the results of salvage radiotherapy trials that showed that prostate bed radiotherapy is efficient in most cases, suggesting that the invisible relapsing cells are mostly located within the prostate bed (4, 5). In cases of biochemical relapse, it is currently recommended that the prostate bed be irradiated before the PSA reaches 0.5 ng/ml (6), as a higher risk of relapse has been shown in patients with more than this threshold (7). Radiotherapy also plays a role in the management of metastatic lymph nodes (8, 9). The NRG Oncology/RTOG 0534 SPPORT Trial showed that irradiating the pelvic lymph nodes in addition to the prostate bed improved progression-free survival compared to the prostate bed alone (10). This important result could be interpreted as radiotherapy being capable not only of reducing local relapses but also of slowing down or even blocking the metastatic process. Altogether, these findings have led to a new appreciation of local and regional control as a determining factor in survival and emphasize the role of combined modality approaches in the treatment of biochemically-relapsing prostate cancer.

Despite adequate salvage prostate bed radiotherapy, retrospective studies suggest that relapses following this technique are still localized in the prostate bed in up to 22% of patients (11–13), which suggests that combatting radioresistance pathways may decrease relapse rates. Moreover, after prostate bed radiotherapy, most relapses occur within the patients’ pelvic lymph nodes (11–13). The dose of radiotherapy to the pelvic lymph nodes is limited by the vicinity of the small bowel. Therefore, the maximum dose to the whole pelvic lymph node is limited to 54-55 Gy in conventional fractionation (14, 15). This dose is lower than the dose usually validated to eradicate microscopic tumor cells within the prostate bed (60 to 66 Gy) (1), which further reinforces the need to biologically escalate the dose to the pelvic lymph nodes by adding radiosensitizing agents. 

To improve the efficacy of post-prostatectomy radiotherapy both locally and distantly, androgen-depriving therapy is one partner of choice as it can radiosensitize prostate cancer cells by both reducing the hypoxic fraction (16) and decreasing testosterone-induced increased DNA repair mechanisms (17, 18). Two major studies combined ADT with radiotherapy to the prostate bed +/- pelvic lymph nodes (4, 5). Both studies showed improved biochemical relapse-free survival, metastasis-free survival and even overall survival in the RTOG 9601 study (4, 5). The recommendation is now to combine ADT and post-prostatectomy radiotherapy in patients with a high risk of biochemical relapse following prostatectomy (6).

Despite combined ADT and irradiation, up to 30% may relapse at 10 years (4, 5). To improve survival, one major area of research is to combine irradiation with active drugs capable of inhibiting micrometastatic cells outside the pelvis, thus improving metastatic control, and ideally also capable of targeting radioresistance pathways to decrease locoregional relapses. The mechanisms behind the aggressiveness and radioresistance of prostate cancer are progressively being revealed. Localized hormone-sensitive prostate cancer is a highly heterogeneous disease (19). Large genomic and transcriptomic studies highlighted several major mechanisms of aggressiveness relating to either the tumor itself (20, 21) or its microenvironment (22, 23), but specific information regarding the genomic mechanisms of radioresistance in prostate cancer is scarce (24, 25). Although several studies have reported histological factors that predict survival after radiotherapy to the intact prostate gland [reviewed in (26, 27)], less information is available on predicting relapse after prostate bed radiotherapy. Recent transcriptomic analyses have described the first test capable of distinguishing low- and high-risk biochemically-relapsing forms of prostate cancer (28). These genomic and transcriptomic analyses can help identify novel mechanisms in prostate cancer progression, and suggest radiosensitizing drugs that target DNA repair, survival pathways, or the tumor microenvironment (29). Based on this better understanding of the mechanisms behind the aggressiveness and radioresistance of prostate cancer, this article describes current and future strategies for drug intensification that aim to improve the efficacy of post-prostatectomy radiotherapy.

### Targeting Microtubule Assembly

In a first attempt to increase the efficacy of prostate bed radiotherapy, multiple studies tried to combine chemotherapeutic agents, such as docetaxel or cabazitaxel, which target microtubule assembly. The combination of docetaxel/prednisone and sunitinib prior to salvage radiotherapy was evaluated in biochemically-relapsing prostate cancer patients (30) (**Table 1**). This study had to be stopped because of excess dose-limiting toxicity (DLT). The progression-free survival rate at 2 years was 51%. Weekly docetaxel combined with prostate bed radiotherapy was better tolerated, but grade 3 neutropenia was noted in almost 50% of patients in a small series of 17 patients (34). At 4 years, progression-free survival was 42% and similar to matched-paired controls. Another taxane derivative, cabazitaxel, was also tested in a Phase II trial (NCT01650285) in combination with radiotherapy after prostatectomy, the results of which are pending. Strategies combining chemotherapy and radiotherapy may result in increased toxicity, with no major improvement in terms of survival.

**Table 1:** Summary of the various molecules currently associated with post-prostatectomy radiotherapy, either in adjuvant situations on anatomopathological criteria (2 studies) or – in most cases – depending on the elevation in PSA after prostatectomy.

Systemic treatment	Study ID	Population	Study Arm	Radiotherapy	Outcomes Results	Recruitment	Results
**mTOR inhibitor**	NCT01548807 Phase I (5-7,5-10mg)	Biochemical recurrence after prostatectomy	Rapamycin + RT	66,6Gy/37F IMRT Daily CBCT	10 mg is safe	Completed	Published (31)
**Abirateron acetate (AA)**	NCT01780220 Phase I,II(CARLHA)	Biochemical recurrence after partial response	A: AA-Prednisolone-ADT-RTB: AA-Prednisolone-RT	Prostate bed radiotherapy IMRT 66Gy/33F	B: not recommended Dose: 750 mg	Completed	Published (19)
**Enzalutamide**	NCT02057939 Phase II( STREAM)	Biochemical relapse after partial response	Enza-ADT-RT	66Gy/33F	2-year PFS: 65%	Completed	Published (20)
**Enzalutamide**	NCT02203695 Phase II (SALV-ENZA)	Biochemical relapse after partial response	A: RT-PlaceboB: RT-Enzalutamide	66,6-70,2 Gy (daily placebo/enzlutamide and 2 months after and before	FFPP (freedom from PSA progression)	Not recruiting	Not published
**Enzalutamide**	NCT03809000 Phase II (STEEL)	Biochemical relapse after prostatectomy	A: Enza-ADT-RTB: ADT-RT	66-70.2 Gy	PFS	Recruiting	Not published
**Apalutamide**	NCT03311555 Phase II(STARTAR)	Biochemical complete response after radical prostatectomy	Apalutamide-ADT-RT + adjuvant cocetaxel	66-74 Gy in 1,8-2 Gy daily fractions over a total of 6-8 weeks	PFS	Not recruiting	Not published
**Apalutamide**	NCT04181203 Phase III (CARLHA-2)	High-risk postprostatectomy biochemically relapsed prostate cancer patients	A: apalutamide-RT-ADTB: RT-ADT	Prostate bed: 66Gy/33FPelvic node: 56,1Gy/33FSIB 69,3/33F to local relapse (TEP/IRM)	PFS	Recruiting	Not published
**Apalutamide**	NCT03899077 Phase II (SAVE)	Biochemical progression after radical prostatectomy	A: ADT-RTB: ADT-RT-Apalutamide	NA	EPIC-26 sexual domain score	Recruiting	Not published
**Apalutamide**	NCT03371719NRG-GU006 Phase II (BALANCE)	Biochemical progression after radical prostatectomy	A: RT-placeboB: RT-apalutamide	NA	bPFS2^nd^: stratification by PAM50 gene expression	Not recruiting	Not published
**AA+Apalutamide**	NCT03141671 Phase II(FORMULA-509)	Rising PSA after prostatectomy with adverse features	A: ADT-AA-apalutamide-RTB: ADT-RT	NA	PFS	Not recruiting	Not published
**Docetaxel Sunitinib**	NCT00734851 Phase II	Rising PSA after prostatectomy	4 cycles D1-D21:Docetaxel-Sunitinib D1-D14	Radiotherapy after docetaxel sunitinib66Gy/33F	PFS	Completed	Published (32)
**Satraplatin**	NCT00480623 Phase I	Rising PSA after prostatectomy	Satraplatin+RT concomittant	NA	DMTDLT	Completed	Not published
**Taxotere**	NCT00480857Phase II	Rising PSA after prostatectomy	Docetaxel 20mg/m2 weekly during RT	64,9-70.3 to tumor bed	No increase in toxicityNo clinical benefit	Completed	Published (33)
**Cabazitaxel**	NCT01650285	Pathological determined stage 3 and/or PSA rising	CabazitaxelDay 1,22,43	64,8 Gy IMRTAdjuvant	DMT	Completed	Not published
**Ixabepilone**	NCT01079793	Pathological determined stage 3	Ixabepilone IB D1-D8D1=D21	IMRTAdjuvant	DMT	Closed	Closed
**Metformin**	NCT02945813 Phase II PROMET	Rising PSA after prostatectomy	A: Metformin 850mg/12h + RTB: RT	70Gy/35F	TTP (time to progression)	Completed	Not published

NA, not assessable.

### Targeting the Androgen Receptor

The androgen receptor (AR) predominantly acts as a transcription factor regulating the expression of genes that maintain cellular homeostasis and normal prostate function (35). Dihydrotestosterone (DHT) binds to the androgen receptor, which then translocates from the cytoplasm to the nucleus, where it binds target genes with an androgen response element (ARE) to provoke a transcriptional response (35). Gene fusions of AR-regulated promoter regions with regions encoding members of the ETS (erythroblast transformation-specific) family of transcription factors are found in 40-60% of localized cases of prostate cancer (36, 37). AR amplification, alternative splicing of the AR, post-translational modifications to the AR, alteration of factors that control AR expression, or somatic gain-of-function mutations which are the hallmark of late-stage castration-resistant prostate cancer, are typically absent in localized cases of prostate cancer (35). This dependence on AR makes localized prostate cancer highly sensitive to blockading AR signaling by drugs that target DHT production, such as LH-RH agonists, or CYP17A1 inhibitors, such as abiraterone acetate, or drugs directly inhibiting the AR such as enzalutamide, darolutamide, or apalutamide.

The interaction between androgens and their receptor triggers intra- and interchromosomal rearrangements by double-stranded breaks. The fusion genes generated this way first initiate, and then promote, prostate cancer (38). This is the case, for example, for the TMPRSS2-ERG fusion gene, which results from a rearrangement mediated by topoisomerase 2B, itself regulated by the androgen receptor pathway (39). However, radiotherapy increases the expression of androgen receptors and stimulates their activity both *in vitro* and *in vivo*, where an increase in the level of hK2, a protein involved in the androgen pathway, is measured in the serum of patients treated with radiotherapy (32). In addition, upregulation of the TMPRSS2 gene mentioned above is observed in the irradiated prostate cell lines. It is therefore understood that radiotherapy may stimulate the androgen receptor pathway, which is thwarted by androgen suppression. Moreover, AR targeting impairs DNA double-strand break repair by inhibiting non-homologous end joining (17, 18), thereby increasing the radiosensitivity of prostate cancer cells.

Two randomized phase 3 studies, GETUG 16 and RTOG 9601, showed there was a strong clinical benefit to combining ADT and prostate bed radiotherapy (4, 5). As preclinical experiments showed that AR-targeting drugs such as enzalutamide are better radiosensitizers than ADT (33), it is expected that combining novel AR-targeting agents may increase the benefits of salvage radiotherapy. Several phase 1 and phase 2 trials have already investigated the combination of radiotherapy and second-generation hormone therapy: abiraterone acetate (AA), enzalutamide, apalutamide, and darolutamide (**Table 1**). The phase 1 CARLHA study was the first to combine salvage prostate bed radiotherapy and AA with or without LH-RH agonists (31). When AA was administered without LH-RH agonists, only 78% achieved castration levels. AA combined with SRT and goserilin did not increase pelvic toxicity but led to an unexpectedly high frequency of grade 3 liver toxicity. The phase II recommended dose of AA combined with goserelin and SRT was 750 mg. Phase 2 results are pending. Enzalutamide was also evaluated in combination with salvage prostate bed radiotherapy (40). Grade 3 toxicities, mostly fatigue and hypertension, were observed in 29% of patients. After a median follow-up time of 37.5 months, 2-year progression-free survival was 65%. Other phase 2 studies are ongoing or closed to accrual. The randomized phase 3 study that compares salvage prostate bed and lymph node radiotherapy combined with 6 months of ADT +/- apalutamide, CARLHA 2 GETUG 33 (NCT04181203), is actively recruiting patients.

### Targeting the Pi3K/Akt/mTOR Pathway

The mammalian target of rapamycin (mTOR) is a protein central to the regulation of cell metabolism and proliferation. mTOR is a downstream effector in the phosphatidylinositol 3-kinase/protein kinase B PI3K/AKT pathway, which regulates metabolism, protein synthesis, growth, cell cycle progression, and survival (41). The Pi3K/AKT pathway is the most frequently activated intra-cellular signaling pathway in prostate cancer, responsible for important signals for malignant transformation, tumor progression, and metastatic invasion. PI3K/AKT is negatively regulated by the PTEN tumor suppressor (phosphatase and tensin homolog) and PTEN deletions are observed in up to 20% of localized cases of prostate cancer. The PI3K­AKT crosstalk with the androgen receptor (AR) pathway and AR signaling blockade results in compensatory activation of the PI3K­AKT pathway (42). The PI3K­AKT pathway also has close links with the RAS/RAF/MEK/ERK pathway and with VEGF (41).

The Pi3K/Akt/mTOR pathway plays a key role in radioresistance through different mechanisms: increased metabolism and proliferation (43), increased DNA repair as AKT regulates DNA-PK activity (44), and mTOR signaling which also plays a key role in hypoxia-triggered angiogenesis and HIF1alpha overexpression (45).

Given the role of PI3K and mTOR in the response of prostate cancer cells to radiation and hypoxia, preclinical studies investigated whether PI3K/AKT/mTOR inhibitors radiosensitized prostate cancer cells of different PTEN status. Several drugs targeting the PI3K/AKT pathway have been developed: Pi3K inhibitors (LY294002, WORTMANNIN, BKM120, GSK2636771), AKT inhibitors (Palomid 539, erufosine, perifosine, ipatasertib), mTOR inhibitors (sirolimus, temsirolimus, everolimus) and dual PI3K/mTOR inhibitors (BEZ235, PI103, GDC-0980). The radiosensitizing properties of these agents have been investigated in several studies in prostate cancer models (46–48). Because of the non-selective profiles of certain drugs in this family, especially with regard to DNA repair, the combination may however be toxic.

The combination of the mTOR inhibitor everolimus and prostate bed radiotherapy without adding ADT was tested in a phase I clinical study (49). The maximum tolerated dose of everolimus in combination with fractionated post-prostatectomy radiation therapy was 10 mg daily, leading to no unexpected toxicity. An undetectable prostate-specific antigen nadir was achieved in more than 50% of patients but information on the PTEN status of responding patients was lacking. With the promising results of ipatasertib in metastatic castration-resistant prostate cancer (50), this area of research warrants further investigation.

### Targeting DNA Repair Pathways

DNA damage response (DDR) genes play an important role in prostate cancer. Men with germline *BRCA2* mutations have a higher risk of aggressive prostate cancer because of MYC activation in combination with inactivation of TP53 and PTEN (51), leading to worse clinical outcomes (52). DDR also plays a major role in the response to radiotherapy, where DNA double strand-breaks are mostly repaired by homologous recombination (HR) and non-homologous end joining (NHEJ). Drugs targeting DDR are very potent radiosensitizers but may increase the likelihood of normal tissue toxicity. In cells lacking efficient HR, such as BRCA2-deficient cells, other DNA repair pathways, such as base excision repair (BER), are responsible for high-fidelity DDR. Combining inhibitors of BER such as polyADP ribosylpolymerase (PARP) inhibitors may therefore radiosensitize HR-deficient cells while not radiosensitizing normal cells with regular HR function. Several PARPi, such as veliparib, olaparib, rucaparib, or talazoparib, were tested in combination with irradiation in prostate cancer models [reviewed in (53)]. Encouraging results suggest increased radiosensitization and limited combined toxicity. To date, no clinical studies have currently addressed the combination of PARPi or any drug targeting DDR in combination with salvage prostate bed radiotherapy.

### Targeting Hypoxia and Glucose Metabolism

Many primary tumors have low levels of molecular oxygen. Hypoxia plays a role in both dissemination, by increasing the genes involved in metastasis, and angiogenesis (e.g. VEGF), and hypoxic tumors respond poorly to radiotherapy. Prostate cancer is strongly hypoxic (54, 55) and a hypoxic signature is a predictor of poor response to radiotherapy (22, 23). The oxic status of the prostate bed following prostatectomy has not been explored, but it is expected that lack of vasculature will render residual tumor cells hypoxic. There are several mechanisms to combat hypoxia: increase oxygen with hyperbaric oxygen or use hypoxic cell radiosensitizers such as tirapazamine for example. Another mechanism is to reduce glucose consumption to force aerobic cells to consume more glucose and reduce glucose concentrations in lesser perfused cells. One of the molecules studied in this sense is metformin, which has already shown its clinical value in retrospective series (56). The SAKK 08/15 – PROMET/GETUG 34 trial (NCT02945813) is a randomized phase II trial that closed recently. It tested salvage radiotherapy +/- metformin in patients with prostate cancer relapsing after prostatectomy. Other inexpensive drugs, such as menadione, could be repurposed to serve as novel radiosensitizers in the treatment of hypoxic prostate cancer (57).

## Perspectives

Not all biochemically-relapsing patients are potential candidates for salvage radiotherapy combined with radiosensitizing drugs (58). The median time from the time of PSA level elevation to the occurrence of metastases is 8 years (59). Strengthening prostate bed radiotherapy by adding drugs can be detrimental in fragile patients with a limited risk of dying of their biochemically-relapsing prostate cancer. The overall benefits of ADT combined with SRT are lost in patients with a lower PSA because of ADT-related cardiovascular toxicity (60). A key point is therefore patient selection, and transcriptomic signatures can help select patients at higher risk of relapse following salvage therapy (28). The European Association of Urology high-risk group of patients with a biochemical relapse following prostatectomy (PSA-doubling time less than 1 year or ISUP grade 4–5 tumors) (58) could help both identify the patients who need treatment intensification and in particular define the use of novel agents in addition to RT+/-ADT. To improve patient selection, the NRG GU006 (NCT03371719) trial is stratifying patients based on their transcriptomic signature to evaluate the benefits of apalutamide combined with ADT and prostate bed radiotherapy. Similarly, genomic characterization of the prostate tumor may help select the best radiosensitizing drug, such as DNA repair inhibitors or immunotherapy in DDR-deficient tumors, or AKT pathway inhibitors in PTEN mutated tumors. As the overall prognosis of biochemically-relapsing patients is good, radiosensitizing candidates should present a strong benefits/risk ratio, which precludes toxic combinations such as certain PI3K inhibitors or chemotherapeutic agents. Lastly, improved phenotypic imaging may help better reduce the target volumes and thereby decrease the likelihood of combined toxicity.

## Conclusion

In this review, we have described therapeutic pathways under investigation in the management of patients experiencing biochemical failure after prostatectomy. However, clinical efficacy remains to be demonstrated for many of the molecules and signaling pathways. In the near future, given the large number of trials, the role of AR-targeting agents should be given a significant role in the management of these patients. Although therapeutic control is an essential point, it is important to take the absence of additional toxicity into account in these long surviving patients with few symptoms.

## Data Availability Statement

The original contributions presented in the study are included in the article/supplementary material. Further inquiries can be directed to the corresponding author.

## Author Contributions

AC and SS contributed to conception and design of the study. AC wrote the first draft of the manuscript. SS, PS, IL, and FS wrote sections of the manuscript. All authors contributed to manuscript revision, read, and approved the submitted version.

## Funding

SS: coordinating investigator of the GETUG 33 CARLHA 2 study funded by Janssen. Research grants: Astellas, AstraZeneca. Expertise and advisory boards: Bayer, Ipsen, Bouchara-Recordati, Takeda.

## Conflict of Interest

SS: coordinating investigator of the GETUG 33 CARLHA 2 study funded by Janssen.

The remaining authors declare that the research was conducted in the absence of any commercial or financial relationships that could be construed as a potential conflict of interest.

## Publisher’s Note

All claims expressed in this article are solely those of the authors and do not necessarily represent those of their affiliated organizations, or those of the publisher, the editors and the reviewers. Any product that may be evaluated in this article, or claim that may be made by its manufacturer, is not guaranteed or endorsed by the publisher.

## Abbreviations

DNA, deoxyribonucleic acid; PI3K, phosphatidylinositol 3-kinases; AKT, protein kinase B; mTOR, mammalian target of rapamycin; NRG, cancer clinical cooperative group including the National Surgical Adjuvant Breast and Bowel Project (NSABP), the Radiation Therapy Oncology Group (RTOG), and the Gynecologic Oncology Group (GOG); SPPORT, short term androgen deprivation with pelvic lymph node or prostate bed only radiotherapy; ADT, androgen-depriving therapies; DLT, dose-limiting toxicity; ARE, androgen response element; ETS, erythroblast transformation-specific; AR, androgen receptor; DHT, di-hydrotestosterone; LH-RH, luteinising hormone-releasing hormone; CYP17A1, cytochrome P450 17A1; TMPRSS2, transmembrane protease, serine 2; ERG, ETS-related gene; hK2, hexokinase 2; GETUG, groupe d’étude des tumeurs urogénitales; CARLHA, combined abiraterone, radiotherapy and LH-RH agonists; AA, abiraterone acetate; SRT, salvage radiotherapy; PTEN, phosphatase and tensin homolog; RAS, rat sarcoma virus protein; RAF, rapidly accelerated fibrosarcoma serine/threonine-protein kinase; MEK, mitogen-activated protein kinase; ERK, extracellular signal-regulated kinases; VEGF, vascular endothelial growth factor; DNA-PK, DNA-dependent protein kinase; DDR, DNA damage response; BRCA2, breast cancer 2; BER, base excision repair; PARPi, polyADP ribosylpolymerase inhibitors; SAKK, Die Schweizerische Arbeitsgemeinschaft für Klinische Krebsforschung; PSA, prostate-specific antigen.
